# Causal relationships of obesity on musculoskeletal chronic pain: A two-sample Mendelian randomization study

**DOI:** 10.3389/fendo.2022.971997

**Published:** 2022-08-23

**Authors:** Xiaoqing Chen, Haifeng Tang, Jinding Lin, Rongdong Zeng

**Affiliations:** Department of Orthopaedics, Quanzhou First Hospital Affiliated to Fujian Medical University, Quanzhou, China

**Keywords:** body mass index, waist circumference, hip circumference, waist-to-hip ratio, Mendelian randomization, chronic pain

## Abstract

**Background:**

The association between obesity and musculoskeletal chronic pain has attracted much attention these days; however, the causal relationship between them is uncertain. Hence, this study performed a Mendelian randomization (MR) analysis to investigate the causal effects of body mass index (BMI), waist circumference (WC), hip circumference (HC), and waist-to-hip ratio (WHR) on knee pain, hip pain, and back pain.

**Materials and methods:**

The summary data for obesity and musculoskeletal chronic pain came from the genome-wide association study datasets. Significant and independent (*p* < 5 × 10^−8^; r^2^ < 0.001, kb = 10,000) single-nucleotide polymorphisms were extracted for MR analysis. The inverse variance weighted (IVW) and other methods were used for MR analysis, while sensitivity analyses were conducted to test the reliability and stability.

**Results:**

The positive causal effects of BMI on knee pain (odds ratio (OR) = 1.049; 95% CI: 1.034 to 1.063; *p* = 9.88 × 10^−12^), hip pain (OR = 1.034; 95% CI: 1.024 to 1.044; *p* = 1.38 × 10^−12^), and back pain (OR = 1.022; 95% CI: 1.007 to 1.038; *p* = 0.004) were observed. WC and HC were also positively associated with knee pain (WC: OR = 1.057; 95% CI: 1.041 to 1.072; *p* = 1.54 × 10^−13^; HC: OR = 1.034; 95% CI: 1.017 to 1.052; *p* = 1.32 × 10^−4^) and hip pain (WC: OR = 1.031; 95% CI: 1.020 to 1.042; *p* = 2.61 × 10^−8^; HC: OR = 1.027; 95% CI: 1.018 to 1.035; *p* = 5.48 × 10^−10^) but not back pain. No causal relationship was found between WHR and musculoskeletal chronic pain. The results were robust according to sensitivity tests.

**Conclusions:**

This study revealed that BMI was positively related to knee, hip, and back pain and that WC and HC were positively associated with knee and hip pain, while WHR was not related to any type of musculoskeletal chronic pain.

## Introduction

Obesity is defined as a disproportionate body weight for height with an excessive accumulation of adipose tissue, which has affected over 640 million population around the world and caused a great burden on society (1). According to the classification of the World Health Organization, individuals are classified as obese when their body mass index (BMI) is over 30 kg/m^2^ (2). Previous studies had illustrated that individuals with obesity were at greater risk for many health problems including metabolic syndrome, type 2 diabetes, osteoarthritis, and other diseases as compared with the normal-weight population (3–5). Chronic pain, as defined by the International Association for the Study of Pain, refers to an unpleasant sensory and emotional experience associated with actual or potential tissue damage, containing back pain, musculoskeletal disorders, and neck pain (6, 7). Chronic pain costs US$560 to US$635 billion per year with a prevalence rate of 11% to 40% (8, 9). Therefore, it is of great significance to explore the relationships between these two heavy-burden and costly diseases.

The association between obesity and pain could be controversial, though many studies about their relationship have been performed. A prospective study including 285 patients and 191 volunteers revealed that shoulder pain was associated with obesity, and it would be helpful to treat pain by losing weight moderately (10). Amabile et al. also found that the risk of having low back pain in individuals with higher BMI was nearly twice that of those with lower BMI, and a dose–response relationship was identified between obesity and pain (11). Similarly, a cohort study in Great Britain found that BMI was associated with knee pain, with results that 19.1% of obese participants had pain symptoms (12). However, Sharon et al. performed a cross-sectional analysis including 142 subjects and revealed that no association was observed between joint pain and obesity (13). The reverse conclusions obtained by different studies might be caused by the limitations (small sample size, different races, and other existing confounders and bias) contained in the cohort and cross-sectional studies. Moreover, these studies could only find the correlation but not the causal relationship between obesity and musculoskeletal chronic pain.

Mendelian randomization (MR) is a method that takes single-nucleotide polymorphism (SNP) as instrumental variables (IVs) to explore the causal effect of exposures on outcomes (14). Due to the great development of large studies of genome-wide association studies (GWASs), MR has been a powerful alternative method for causal inference (15). Since genotypes appear before the occurrence of diseases and are largely unrelated to lifestyle or environmental factors after birth, MR could minimize the confounding factors and avoid reverse causality bias (16). This current study aims at detecting the causal effects of BMI, waist circumference (WC), hip circumference (HC), and waist-to-hip ratio (WHR) on knee, hip, and back pain with a two-sample MR method based on GWAS datasets.

## Methods

### Study design and genome-wide association study data source

**Figure 1:**
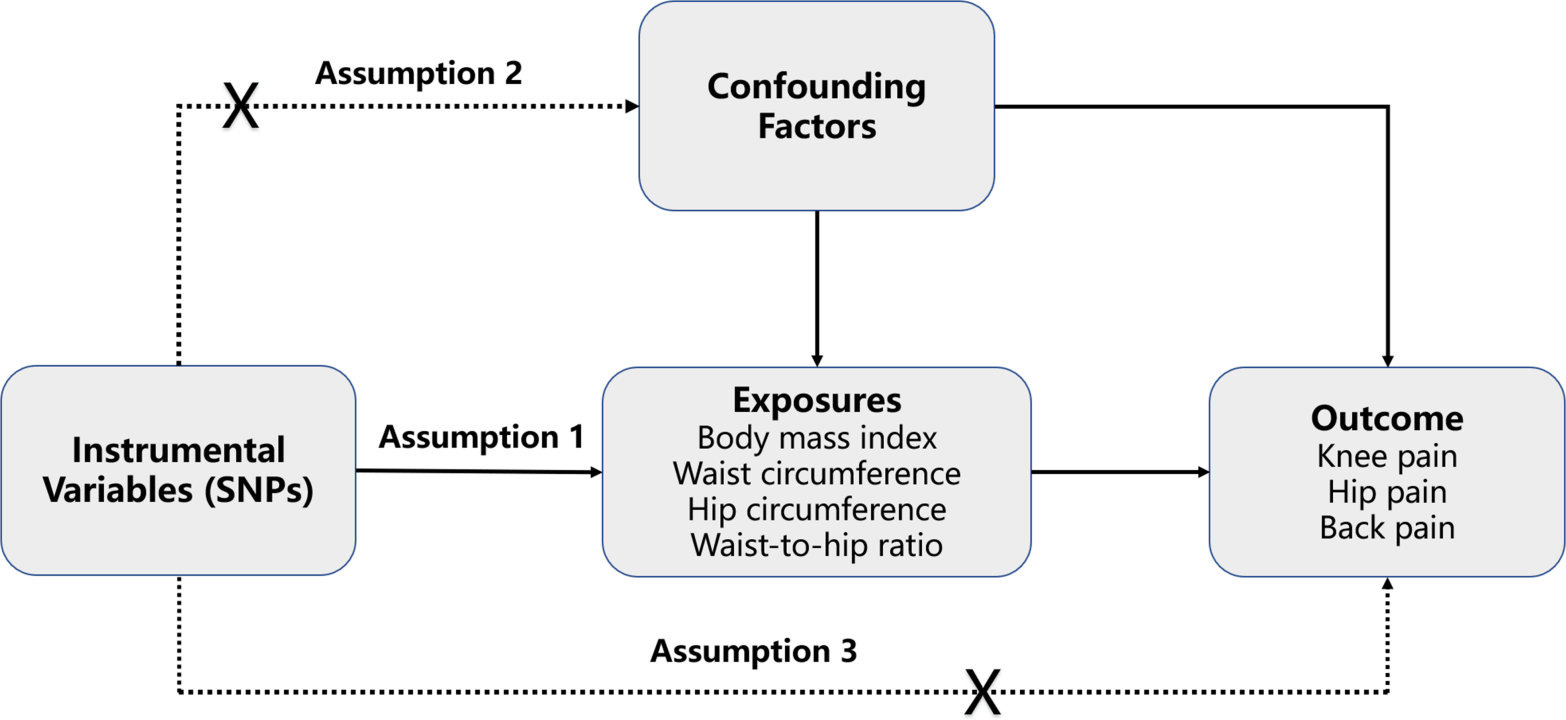
The diagram of Mendelian randomization study. Three assumptions are required. Firstly, the genetic variants should be closely related to the exposures. Secondly, the genetic variants are supposed to be independent of confounding factors. Thirdly, the effects of the genetic variants on outcomes are only mediated by the exposures. SNPs, single-nucleotide polymorphisms.

The diagram of the study design for this MR analysis is shown in **Figure 1**. From the sketch map, we could learn that three assumptions are supposed to be met in the MR analysis. Firstly, the genetic variants should be closely related to the exposures. Secondly, the genetic variants are supposed to be independent of confounding factors. Lastly, the effects of the genetic variants on outcomes are only mediated by the exposures. As mentioned earlier, the SNPs were taken as IVs at a genome-wide significant level (*p* < 5 × 10^−8^) to perform the causal relationships between obesity and pain. Then linkage disequilibrium (LD) was calculated among the selected SNPs, and the SNPs with LD (while r^2^ > 0.001 or physical distance between them was within 10,000 kb) were excluded. Moreover, the *F*-statistics were also calculated, and weak IVs were removed (*F* < 10). The Steiger filtering test was also performed to exclude SNPs that explain more of the variance in the outcome rather than exposure.

The data used in the MR analyses originated from the GWAS summary data. Genetic data for BMI traits came from the GIANT consortium, which included 322,154 individuals and 2,554,668 SNPs of European ancestry (17). The genetic variants for waist circumference (N = 232,101), hip circumference (N = 213,038), and waist-to-hip ratio traits (N = 212,244) also originated from European population based on the GIANT consortium (18). Additionally, as for the outcomes, genetic variants for knee pain conducted by Ben et al. contained 98,704 cases and 363,153 controls from UK Biobank sources. The datasets for hip pain (52,087 patients and 409,770 controls) and back pain (118,471 cases and 343,386 controls) were also obtained from a GWAS meta-analysis of UK Biobank sources. All the genetic data were from patients with European ancestry and could be obtained from publicly available GWAS datasets (https://gwas.mrcieu.ac.uk). The MR-PRESSO test was applied to figure out and remove any potential outliers. Since the data of this study were based on existing publications and public databases, additional ethical approval or consent to participate was not required.

### Statistical analyses

In this MR analysis, inverse variance weighted (IVW), weighted median, and MR-Egger were adopted to assess the causal effects of BMI, WC, HC, and WHR on knee pain, hip pain, and back pain. The IVW method analyzes each Wald ratio and provides a consistent estimate of the causal effect when all instrumental variables are valid, which is mainly used in the results with no heterogeneity or directional pleiotropy (19). The weighted median method was also used, for it could provide consistent estimates when up to 50% of the weight in the analysis originated from invalid instrumental variables (20). It should be noticed that the weighted median method would give a more accurate estimation than IVW when heterogeneity existed in the results. The MR-Egger method could identify and correct potential pleiotropy (*p*-value of intercept <0.05) and gives a consistent estimate (21). However, the effect size rather than the statistical significance of MR-Egger was concentrated in this current study, for the statistical power of MR-Egger was low (22). The results were reported as odds ratio (OR) and 95% confidence intervals (CIs), which provided an estimate of risk for outcome caused by each standard deviation (SD) increase in the risk factor.

### Sensitivity testing

Then sensitivity analyses were conducted for the quality control of the MR results. Cochran’s Q test and I^2^ statistics were used to detect heterogeneity, and the intercept of the MR-Egger and MR-PRESSO tests were used to explore the directional pleiotropy. Additionally, this study performed a “leave-one-out” sensitivity test to evaluate whether the analysis was biased by a single SNP that had a particularly large horizontal pleiotropic effect. All statistical analyses were performed by the “Two-Sample MR” package (version 0.5.6) in R (version 4.1.2) software. The results were considered to be statistically significant when *p* < 0.05 and with heterogeneity when I^2^ > 50%.

## Results

### The extracted single-nucleotide polymorphisms for Mendelian randomization analyses

In this two-sample MR study, BMI, WC, HC, and WHR were taken as risk factors, while knee, hip, and back pain were taken as outcomes. The basic information of relevant SNPs that were selected for MR analyses is listed in **Supplementary Material**. A total of 69 SNPs with a mean of *F* = 66.78 associated with BMI and musculoskeletal chronic pain, 42 SNPs with a mean of *F* = 59.26 associated with WC and musculoskeletal chronic pain, 52 SNPs with a mean of *F* = 54.99 related to HC and musculoskeletal chronic pain, and 29 SNPs with a mean of *F* = 48.34 related to WHR and musculoskeletal chronic pain were included in this study.

### Causal relationship of obesity on knee pain

**Figure 2:**
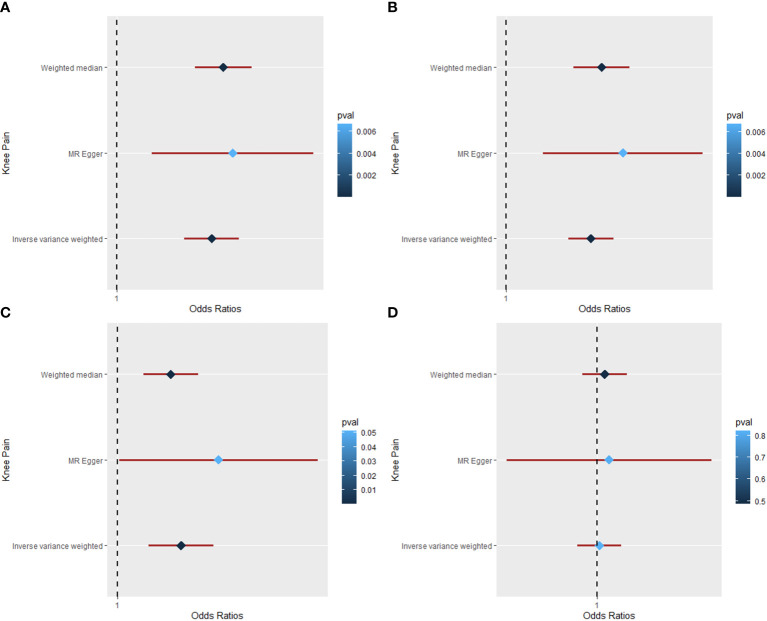
The Mendelian randomization analysis for causal effects of obesity on knee pain. **(A)** Forest plot about the causal effect of body mass index on knee pain. **(B)** Forest plot about the causal effect of waist circumference on knee pain. **(C)** Forest plot about the causal effect of hip circumference on knee pain. **(D)** Forest plot about the causal effect of waist-to-hip ratio on knee pain.

The MR results for the causal effects of BMI, WC, HC, and WHR on knee pain are listed in **Table 1**, **Figure 2**, and **Figures S1–S3**. With one SD increase in BMI, the risk of knee pain was 1.049-fold as analyzed by the IVW method (95% CI: 1.034 to 1.063; *p* = 9.88 × 10^−12^). Similar results could also be acquired by weighted median (OR = 1.055; 95% CI: 1.040 to 1.070; *p* = 2.67 × 10^−13^) and MR-Egger (OR = 1.060; 95% CI: 1.018 to 1.104; *p* = 0.007) methods. Based on Cochran’s Q, I^2^, and MR-Egger intercept tests, there was heterogeneity (Q = 172.46, *p* = 3.04 × 10^−11^; I^2^ = 61.15%) but no pleiotropy (intercept beta = −3.00 × 10^−4^, *p* = 0.593) in the results. Then the “leave-one-out” sensitivity revealed that the causal relationship between BMI and knee pain was not changed by individual SNPs, meaning that the results were stable and reliable (**Figure S1**). The positive causal relationships between WC and knee pain were proved by the IVW (OR = 1.057; 95% CI: 1.041 to 1.072; *p* = 1.54 × 10^−13^), weighted median (OR = 1.064; 95% CI: 1.045 to 1.084; *p* = 2.51 × 10^−11^), and MR-Egger (OR = 1.079; 95% CI: 1.024 to 1.137; *p* = 0.007) methods. However, heterogeneity (Q = 72.40, *p* = 0.001) was observed in this causal relationship. No pleiotropy (intercept beta = −5.90 × 10^−4^, *p* = 0.416) was found in the results according to the MR-Egger intercept and MR-PRESSO tests. In the “leave-one-out” analysis, no single SNP strongly drove the overall effect of WC on knee pain (**Figure S2**).

**Table 1:** The MR results regarding causal associations between obesity and chronic pain.

Outcome	Exposure	Method	SNP (n)	OR	95% CI	*p*-Value
**Knee pain**	BMI	MR-Egger	69	1.060	1.018, 1.104	0.007
		Inverse variance weighted	69	1.049	1.034, 1.063	9.88 × 10^−12^
		Weighted median	69	1.055	1.040, 1.070	2.67 × 10^−13^
	WC	MR-Egger	42	1.079	1.024, 1.137	0.007
		Inverse variance weighted	42	1.057	1.041, 1.072	1.54 × 10^−13^
		Weighted median	42	1.064	1.045, 1.084	2.51 × 10^−11^
	HC	MR-Egger	52	1.055	1.001, 1.112	0.051
		Inverse variance weighted	52	1.034	1.017, 1.052	1.32 × 10^−4^
		Weighted median	52	1.029	1.014, 1.044	1.34 × 10^−4^
**Hip pain**	BMI	MR-Egger	69	1.039	1.010, 1.068	0.009
		Inverse variance weighted	69	1.034	1.024, 1.044	1.38 × 10^−12^
		Weighted median	69	1.034	1.023, 1.045	1.60 × 10^−9^
	WC	MR-Egger	42	1.064	1.026, 1.104	0.002
		Inverse variance weighted	42	1.031	1.020, 1.042	2.61 × 10^−8^
		Weighted median	42	1.031	1.018, 1.045	5.04 × 10^−6^
	HC	MR-Egger	52	1.051	1.026, 1.077	2.11 × 10^−4^
		Inverse variance weighted	52	1.027	1.018, 1.035	5.48 × 10^−10^
		Weighted median	52	1.025	1.013, 1.038	7.03 × 10^−5^
**Back pain**	BMI	MR-Egger	69	1.029	0.984, 1.076	0.219
		Inverse variance weighted	69	1.022	1.007, 1.038	0.004
		Weighted median	69	1.017	1.002, 1.032	0.023
	WC	MR-Egger	42	1.030	0.963, 1.102	0.397
		Inverse variance weighted	42	1.027	1.007, 1.046	0.006
		Weighted median	42	1.011	0.993, 1.029	0.234
	HC	MR-Egger	52	1.028	0.982, 1.077	0.244
		Inverse variance weighted	52	1.021	1.006, 1.036	0.007
		Weighted median	52	1.011	0.995, 1.027	0.194

SNPs, single-nucleotide polymorphisms; OR, odds ratios; CI, confidence interval; BMI, body mass index; WC, waist circumference; HC, hip circumference.

As for the HC trait, both IVW (OR = 1.034; 95% CI: 1.017 to 1.052; *p* = 1.32 × 10^−4^) and weighted median (OR = 1.029; 95% CI: 1.014 to 1.044; *p* = 1.34 × 10^−4^) methods showed positive causal effects of HC on knee pain. There was no pleiotropy (intercept beta = −6.3 × 10^−4^, *p* = 0.436), but heterogeneity (Q = 202.08, *p* = 7.22 × 10^−20^; I^2^ = 74.76%) was observed in the results based on Cochran’s Q, I^2^, and MR-Egger intercept tests. Then the “leave-one-out” analysis was conducted, revealing that the positive causal relationship was stable and reliable (**Figure S3**). The MR analysis for the causal relationship of WHR on knee pain was also conducted; however, no causal effect was found between them by IVW (OR = 1.003; 95% CI: 0.979 to 1.027; *p* = 0.822), weighted median (OR = 1.009; 95% CI: 0.984 to 1.034; *p* = 0.486), and MR-Egger (OR = 1.014; 95% CI: 0.904 to 1.136; *p* = 0. 816) methods.

### Causal relationship of obesity on hip pain

**Table 1**, **Figure 3**, and **Figures S4–S6** show the MR results of causal relationships between BMI, WC, HC, WHR, and hip pain. According to the results in **Table 1**, there was a positive causal effect of BMI on hip pain as analyzed by IVW (OR = 1.034; 95% CI: 1.024 to 1.044; *p* = 1.38 × 10^−12^), weighted median (OR = 1.034; 95% CI: 1.023 to 1.045; *p* = 1.60 × 10^−9^), and MR-Egger (OR = 1.039; 95% CI: 1.010 to 1.068; *p* = 0. 009) methods. Heterogeneity (Q = 132.53, *p* = 3.29 × 10^−6^) but not pleiotropy (intercept beta = −1.30 × 10^−4^, *p* = 0.739) was found in the MR results by Cochran’s Q and MR-Egger intercept tests. The “leave-one-out” analysis revealed that the result was stable and reliable, for no single SNP would influence the results (**Figure S4**). Additionally, with one SD higher than WC, the risk of hip pain increased by approximately 3.1% by the IVW (95% CI: 1.020 to 1.042; *p* = 2.61 × 10^−8^) and weighted median (95% CI: 1.018 to 1.045; *p* = 5.04 × 10^−6^) methods, while the risk increased by nearly 6.4% according to the MR-Egger (95% CI: 1.026 to 1.104; *p* = 0.002) method. However, heterogeneity (Q = 63.87, *p* = 0.010) existed in the results based on the Cochran’s Q test. The MR-Egger intercept and “leave-one-out” tests revealed that there was no pleiotropy (intercept beta = −9.00 × 10^−4^, *p* = 0.082) or single SNP that strongly drove the overall effect (**Figure S5**), which means that the result was robust.

**Figure 3:**
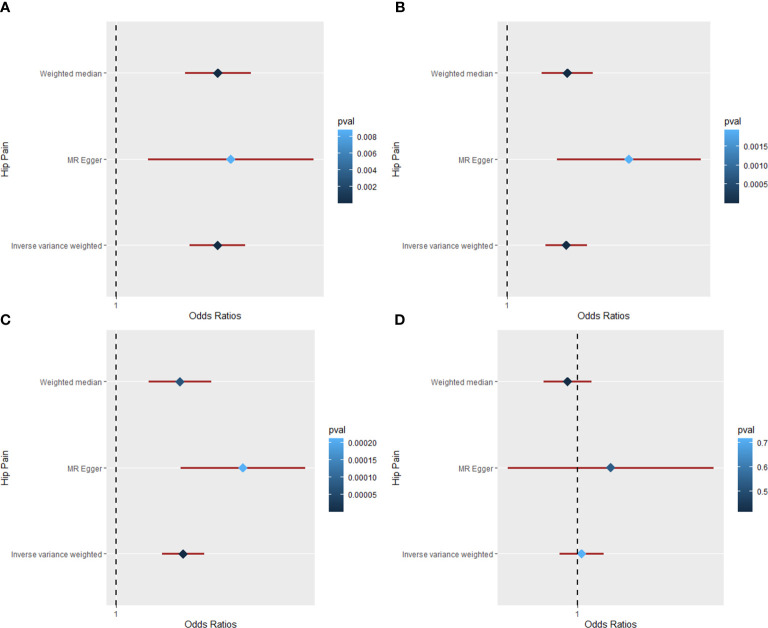
The Mendelian randomization analysis for causal effects of obesity on hip pain. **(A)** Forest plot about the causal effect of body mass index on hip pain. **(B)** Forest plot about the causal effect of waist circumference on hip pain. **(C)** Forest plot about the causal effect of hip circumference on hip pain. **(D)** Forest plot about the causal effect of waist-to-hip ratio on hip pain.

Moreover, the IVW (OR = 1.027; 95% CI: 1.018 to 1.035; *p* = 5.48 × 10^−10^), weighted median (OR = 1.025; 95% CI: 1.013 to 1.038; *p* = 7.03 × 10^−5^), and MR-Egger (OR = 1.051; 95% CI: 1.026 to 1.077; *p* = 2.11 × 10^−4^) methods all showed a positive causal effect of HC on hip pain (**Table 1**). According to Cochran’s Q and MR-Egger intercept tests, there was heterogeneity (Q = 77.63, *p* = 0.010) but no pleiotropy (intercept beta = −7.5 × 10^−4^, *p* = 0.051) that existed in the MR result. The “leave-one-out” analysis illustrated that no single SNP strongly drove the overall effect between HC and hip pain (**Figure S6**). Additionally, the causal relationship between WHR and hip pain was also studied in this study. However, all the three methods yield a null causal relationship between them (IVW: OR = 1.003; 95% CI: 0.986 to 1.021; *p* = 0.717; weighted median: OR = 0.992; 95% CI: 0.974 to 1.011; *p* = 0.414; MR-Egger: OR = 1.027; 95% CI: 0.946 to 1.114; *p* = 0.533). No significant pleiotropy was detected in the analysis, and the “leave-one-out” test revealed a stable and reliable result.

### Causal relationship of obesity on back pain

**Figure 4:**
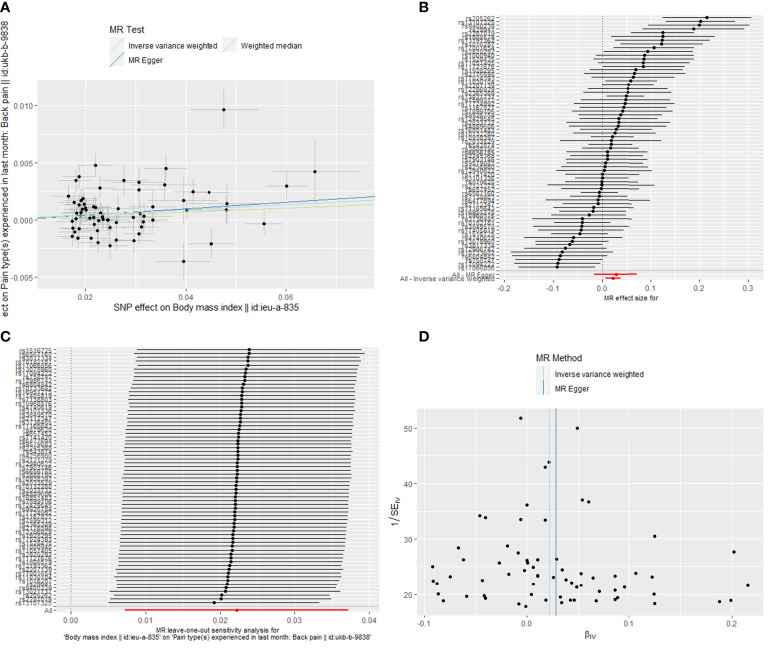
The Mendelian randomization analysis for causal effects of body mass index on back pain. **(A)** Scatter plot about the causal effect of body mass index on back pain. **(B)** Forest plot for the overall causal effects of body mass index on back pain. **(C)** Leave-one-out analysis for the causal effect of body mass index on back pain. **(D)** Funnel plot of SNPs related to body mass index and back pain. SNPs, single-nucleotide polymorphisms.

The MR results of causal relationships of BMI, WC, HC, and WHR on back pain are shown in **Table 1** and **Figure 4**. With one SD increase in BMI trait, the incidence of back pain raised nearly 2% as analyzed by IVW (OR = 1.022; 95% CI: 1.007 to 1.038; *p* = 0.004) and weighted median (OR = 1.017; 95% CI: 1.002 to 1.032; *p* = 0.023) method. The results of Cochran’s Q and I^2^ tests showed the existence of heterogeneity (Q = 182.95, *p* = 1.06 × 10^−12^; I^2^ = 63.38). No pleiotropy (intercept beta = −1.7 × 10^−4^, *p* = 0.779) was observed in the MR analysis. In the “leave-one-out” analysis, no single SNP strongly drove the overall causal effect of BMI on back pain (**Figure 4**). As for the relationships between WC, HC, and back pain, causal effects were found among them by IVW (WC: OR = 1.027; 95% CI: 1.007 to 1.046; *p* = 0.006; HC: OR = 1.021; 95% CI: 1.006 to 1.036; *p* = 0.007). However, the weighted median (WC: OR = 1.011; 95% CI: 0.993 to 1.029; *p* = 0.234; HC: OR = 1.011; 95% CI: 0.995 to 1.027; *p* = 0.194) and MR-Egger (WC: OR = 1.030; 95% CI: 0.963 to 1.102; *p* = 0.397; HC: OR = 1.028; 95% CI: 0.982 to 1.077; *p* = 0.244) methods both showed a null causal effect of WC and HC on back pain. Due to the existence of heterogeneity but no pleiotropy, the weighted median method was adopted in the MR analysis; hence, the conclusions of causal effects among them were dropped. Additionally, the results revealed a null causal association between WHR and back pain by IVW (OR = 0.996; 95% CI: 0.975 to 1.018; *p* = 0.733). Weighted median and MR-Egger were consistent with the IVW method.

## Discussion

This current research explored the causal relationships between obesity and musculoskeletal chronic pain based on the GWAS summary datasets, which might be the first study to investigate the causal effects of BMI, WC, HC, and WHR on knee, hip, and back pain by MR analysis. It revealed that BMI was positively related to knee, hip, and back pain and that WC and HC were positively associated with knee and hip pain, while WHR was not related to any type of musculoskeletal chronic pain.

Many previous studies have revealed the conclusion that being overweight or extremely obese could be associated with musculoskeletal chronic pain. In the United States, a survey concerning over 1 million individuals revealed that BMI was positively associated with daily pain and that obese people had a higher risk of being affected by daily pain (23). Another population-based longitudinal study containing 7,977 participants conducted by Rahman et al. in Finland found that central obesity defined by waist circumference was positively related to low back pain (OR = 1.40; 95% CI; 1.16 to 1.68), while general obesity measured with body mass index was positively associated with lumbar radicular pain (OR = 1.44; 95% CI: 1.12 to 1.85) (24). Similarly, Min et al. performed a research involving 40,999 individuals among community-dwelling older adults, showing that both overweight (OR = 1.166; 95% CI: 1.104 to 1.232, *p* < 0.01) and obesity (OR = 1.786; 95% CI: 1.530 to 2.085, *p* < 0.01) significantly contributed to the musculoskeletal chronic pain (25). Additionally, in another cross-sectional study with 6,524 elderly individuals in China, the researchers illustrated that the participants in overweight and obesity groups were more likely to be suffering from musculoskeletal chronic pain, compared with the normal-weight groups (26). The conclusions of these studies were consistent with our results that BMI and other obesity-related traits could causally increase musculoskeletal chronic pain.

On the contrary, there were also many scholars who found that obesity was not related to musculoskeletal chronic pain. An observational study on adolescent girls found that BMI was not associated with pain tolerance (27). Also, Paula et al. conducted a cross-sectional study containing 690 participants based in Brazil to investigate the relationship between obesity (evaluated by body mass index, bioimpedance, skinfold, arm, and abdominal circumference) and temporomandibular disorder pain in adolescents, and they discovered that all obesity-related traits were not related with temporomandibular disorder pain (28). Additionally, a matched pair study involving 1,128 female twins was performed to explore the relationships between BMI, percent body fat, WC, WHR, and low back pain, and no associations were observed between different obesity measures and low back pain after the full adjustment for genetic factors in this monozygotic within-pair case–control research (29). However, the different conclusions could be attributed to the diverse study designs and the existence of confounding factors as well as bias in observational studies. More importantly, these cross-sectional or cohort studies could only investigate the correlation but not the causal relationships between obesity and musculoskeletal chronic pain.

The association between obesity and musculoskeletal chronic pain could be explained by the mechanical loading and biochemical mechanisms (30). It was supposed that additional weight would give more pressure on the weight-bearing joints and skeletal muscle. Geoffrey et al. performed a study aiming at exploring different kinds of structural failures in intervertebral discs and found that chronic loadings would lead to a severely damaging impact on the spine, which could cause back pain (31). Similarly, another study was performed to detect the biomechanical stresses of the lower back through weight lifting tasks; it revealed that obese participants had a higher risk for back pain caused by the damage of lifting objects as compared with the normal-weight group (32). Moreover, inflammation also played a crucial part in uncovering the potential mechanism of the causal association between obesity and pain. It was widely known that obesity was considered a low-grade inflammatory disease and could produce many cytokines and adipokines through adipose tissue, which might be associated with musculoskeletal chronic pain (33). Some studies illustrated that leptin, one of the adipokines related to obesity, could increase the production of proteases and nitric oxide so as to cause low back pain (34). Another research revealed that galanin could regulate the pain threshold in obesity by central galanin receptor-1 and peripheral galanin receptor-2, though the antinociceptive effect of activating the receptors had not been clearly characterized (35). These theories could briefly explain the inner association between obesity musculoskeletal chronic pain, while more studies are need for the underlying mechanisms.

As for this Mendelian randomization analysis, there are many strengths that should be mentioned. This study investigated the causal relationships between obesity and knee, hip, and back pain with a large sample size based on GWAS datasets, which provided great persuasion for the associations. This current MR study could minimize the effects of bias and confounding factors due to the genetic variant alleles assigned randomly. Moreover, the reverse causality between obesity and musculoskeletal chronic pain that existed in the cross-sectional or cohort studies could also be avoided in this MR study, for the genetic variant exhibits earlier than diseases. Nevertheless, the limitations are also included in this study. Firstly, this MR analysis was based on patients of European ancestry, causing the obtained results to be boundedness, meaning that other races might have different results. Additionally, it was difficult for us to make further subgroup analyses for the causal effects of obesity on other sites or the degree of musculoskeletal chronic pain due to the restrictions of original data.

In conclusion, this present study found positive causal associations between BMI on knee pain, hip pain, and back pain. The waist circumference and hip circumference were also positively associated with knee pain and hip pain. However, no causal relationship was found between waist-to-hip ratio and knee, hip, and back pain. This research could provide much help for the potential mechanism and weight management in obese population with musculoskeletal chronic pain.

## Data availability statement

The original contributions presented in the study are included in the article/**Supplementary Material**. Further inquiries can be directed to the corresponding author.

## Ethics statement

Since the data of this study were based on existing publications and public databases, additional ethical approval or consent to participate was not required.

## Author contributions

XC performed the study and wrote the manuscript. HT and JL interpreted the results. RZ designed this study. All the authors contributed to the article and approved the submitted version.

## Funding

The present study was supported by the Natural Science Foundation of Fujian Province (2022J011457) and Quanzhou Science and Technology Plan Project (2021N061S).

## Acknowledgments

We thank the developers of the GWAS datasets.

## Conflict of interest

The authors declare that the research was conducted in the absence of any commercial or financial relationships that could be construed as a potential conflict of interest.

## Publisher’s note

All claims expressed in this article are solely those of the authors and do not necessarily represent those of their affiliated organizations, or those of the publisher, the editors and the reviewers. Any product that may be evaluated in this article, or claim that may be made by its manufacturer, is not guaranteed or endorsed by the publisher.

## Abbreviations

BMI, body mass index; MR, Mendelian randomization; SNP, single-nucleotide polymorphism; IVs, instrumental variables; GWAS, genome-wide association study; WC, waist circumference; HC, hip circumference; WHR, waist-to-hip ratio; LD, linkage disequilibrium; IVW, inverse variance weighted; OR, odds ratio; CIs, confidence intervals; SD, standard deviation.
