# Supplementary material for: Causal relationships of obesity on musculoskeletal chronic pain: A two-sample Mendelian randomization study
Source: Front Endocrinol (Lausanne). 2022 Aug 23;13:971997. doi: 10.3389/fendo.2022.971997 (PMC9445165; doi:10.3389/fendo.2022.971997)
Supplement: Supplementary file 1 [file DataSheet_1.pdf]

Supplementary Figures

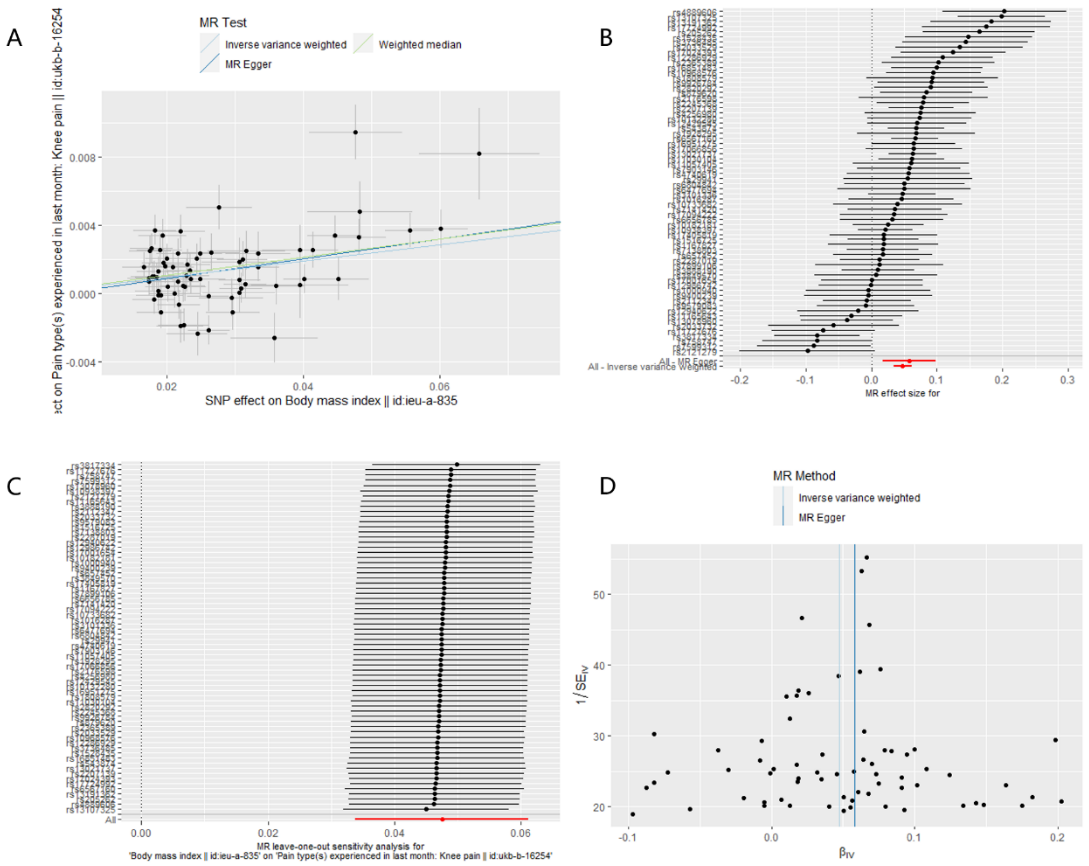

Figure S1 The Mendelian Randomization analysis for causal effects of body mass index on knee pain. A: Scatter plot about the casual effect of body mass index on knee pain; B: Forest plot for the overall causal effects of body mass index on knee pain; C: Leave-one-out analysis for the casual effect of body mass index on knee pain; D: Funnel plot of SNPs related to body mass index and knee pain.

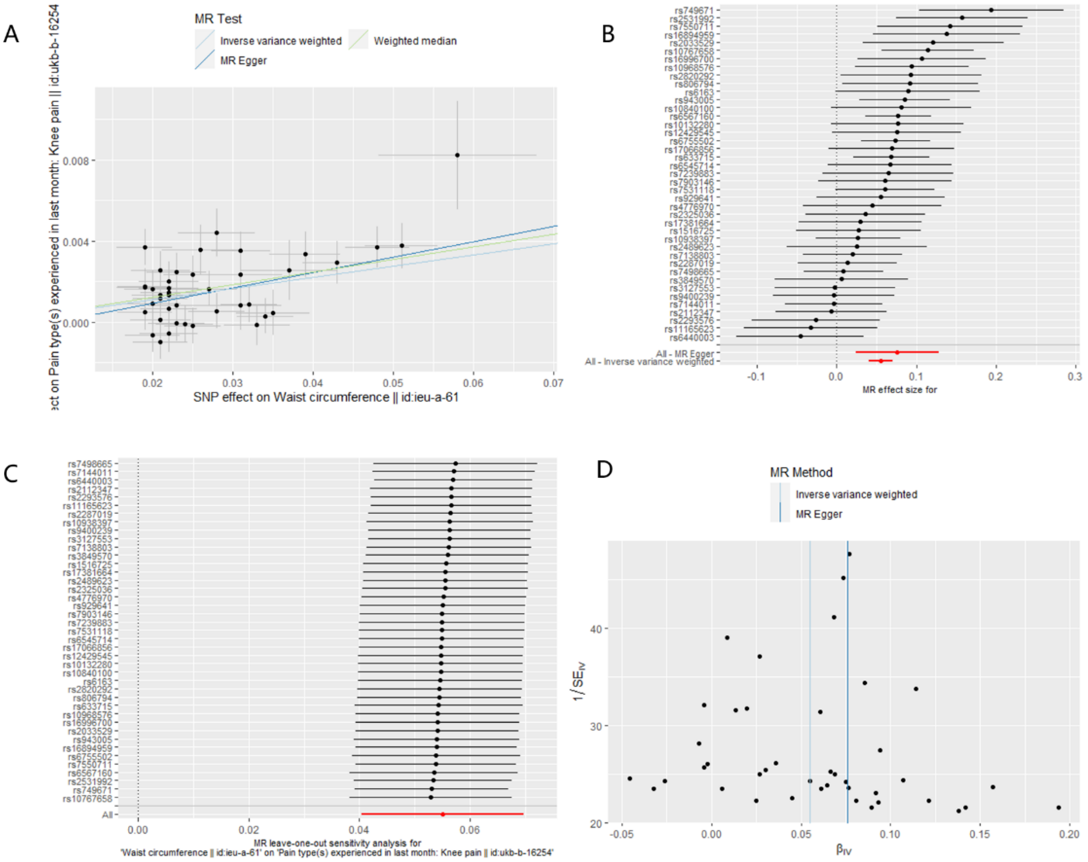

Figure S2 The Mendelian Randomization analysis for causal effects of waist circumference on knee pain. A: Scatter plot about the casual effect of waist circumference on knee pain; B: Forest plot for the overall causal effects of waist circumference on knee pain; C: Leave-one-out analysis for the casual effect of waist circumference on knee pain; D: Funnel plot of SNPs related to waist circumference and knee pain.

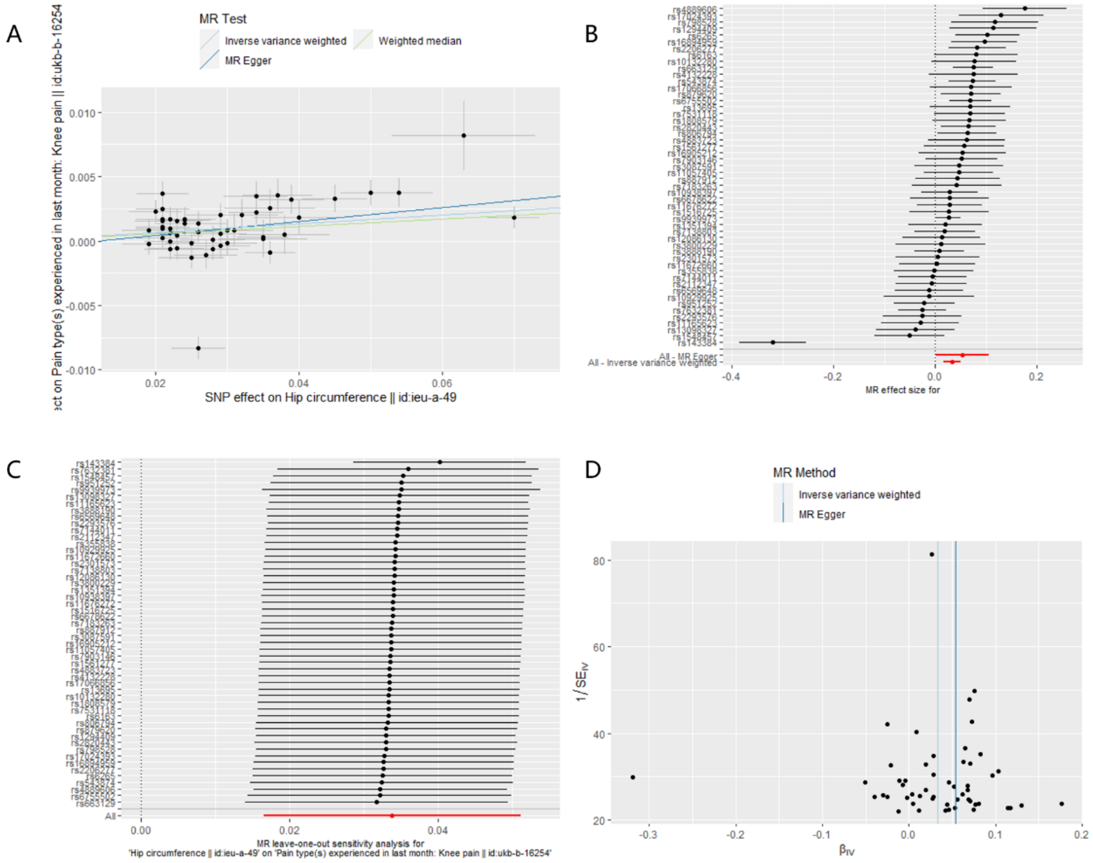

Figure S3 The Mendelian Randomization analysis for causal effects of hip circumference on knee pain. A: Scatter plot about the casual effect of hip circumference on knee pain; B: Forest plot for the overall causal effects of hip circumference on knee pain; C: Leave-one-out analysis for the casual effect of hip circumference on knee pain; D: Funnel plot of SNPs related to hip circumference and knee pain.

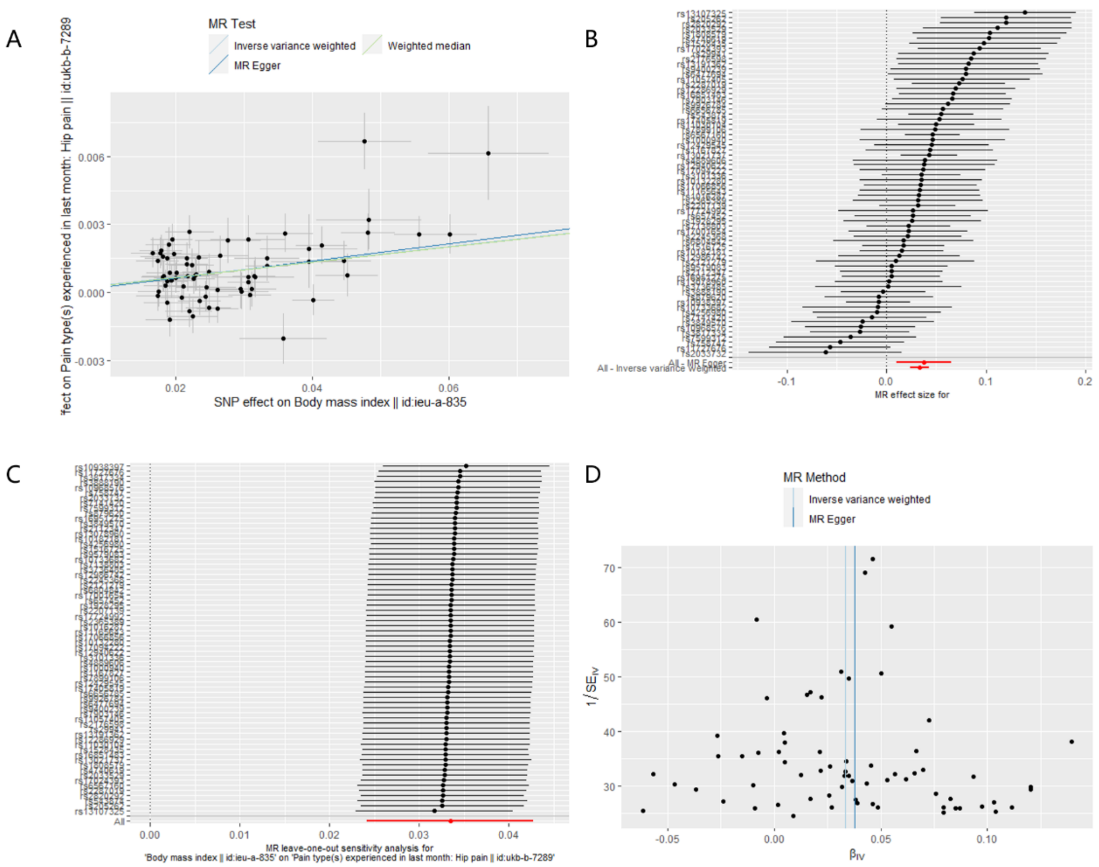

Figure S4 The Mendelian Randomization analysis for causal effects of body mass index on hip pain. A: Scatter plot about the casual effect of body mass index on hip pain; B: Forest plot for the overall causal effects of body mass index on hip pain; C: Leave-one-out analysis for the casual effect of body mass index on hip pain; D: Funnel plot of SNPs related to body mass index and hip pain.

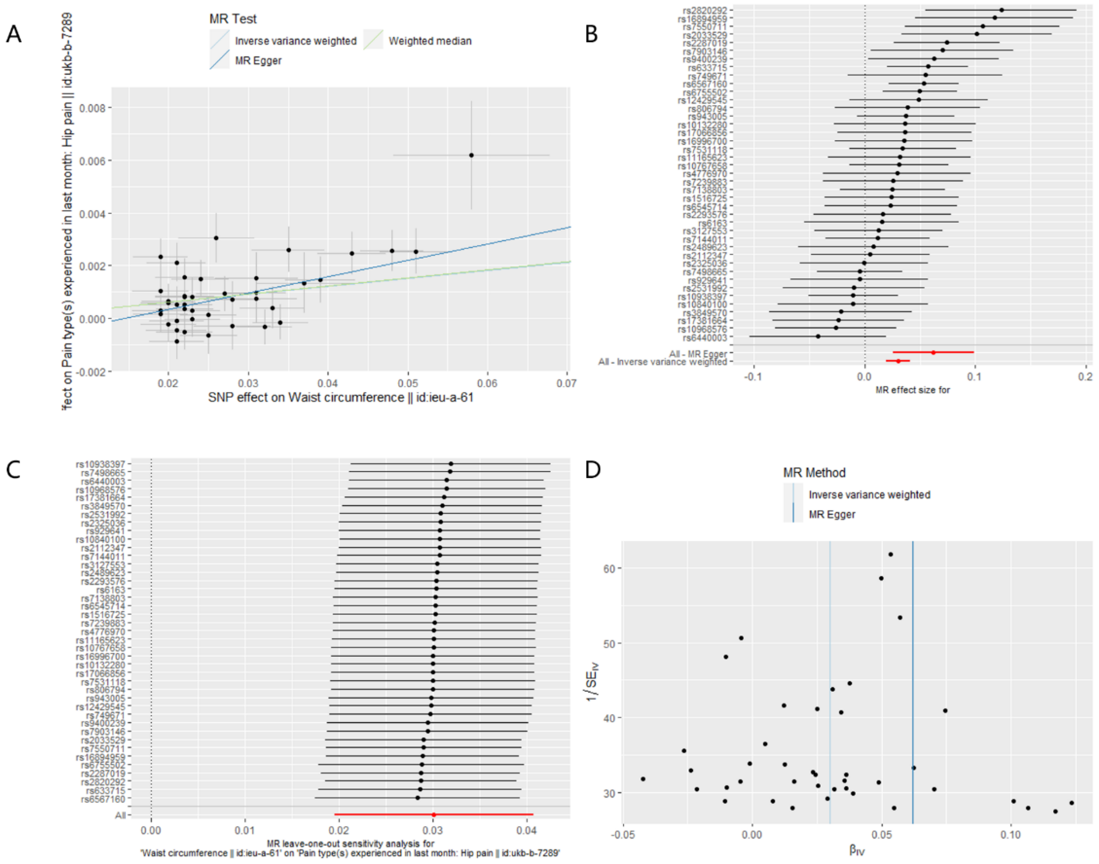

Figure S5 The Mendelian Randomization analysis for causal effects of waist circumference on hip pain. A: Scatter plot about the casual effect of waist circumference on hip pain; B: Forest plot for the overall causal effects of waist circumference on hip pain; C: Leave-one-out analysis for the casual effect of waist circumference on hip pain; D: Funnel plot of SNPs related to waist circumference and hip pain.

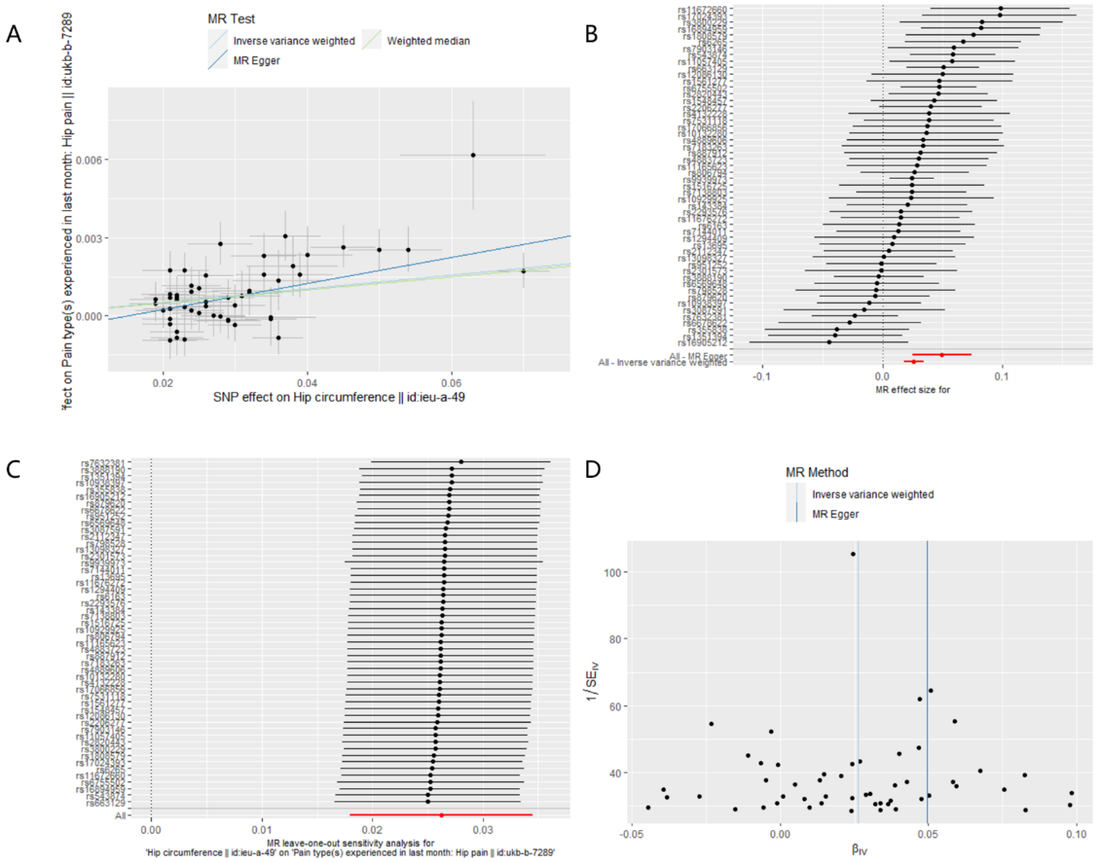

Figure S6 The Mendelian Randomization analysis for causal effects of hip circumference on hip pain. A: Scatter plot about the casual effect of hip circumference on hip pain; B: Forest plot for the overall causal effects of hip circumference on hip pain; C: Leave-one-out analysis for the casual effect of hip circumference on hip pain; D: Funnel plot of SNPs related to hip circumference and hip pain.
